# Neural circuits revealed

**DOI:** 10.3389/fncir.2015.00035

**Published:** 2015-07-28

**Authors:** Mariano Soiza-Reilly, Peter Saggau, Benjamin R. Arenkiel

**Affiliations:** ^1^Institut du Fer à Moulin (U839), INSERM/Université Pierre et Marie CurieParis, France; ^2^Allen Institute for Brain ScienceSeattle, WA, USA; ^3^Department of Molecular and Human Genetics, Baylor College of MedicineHouston, TX, USA

**Keywords:** neuronal tracing, ultrastructure, connectome, optogenetics, networks, electrophysiology, behavior, neurological and psychiatric disorders

The appropriate function of the nervous system relies on precise patterns of connectivity among hundreds to billions of neurons across different biological systems. Evolutionarily conserved patterns of neural circuit organization and connectivity between morphologically and functionally diverse sets of neurons emerge from a remarkably robust set of genetic blueprints, uniquely defining circuits responsible for planning and execution of behavioral repertoires (Arenkiel et al., 2004; Dasen, 2009; Pecho-Vrieseling et al., 2009; Sürmeli et al., 2011; White and Sillitoe, 2013; Inamata and Shirasaki, 2014). Although it is well-established that individual neurons represent the elemental building blocks of the brain, understanding the architecture of neural circuits and how neurons functionally “wire up” through synapses, remains one of biology's major challenges. Our current understanding of how interconnected neuronal populations produce perception, memory, and behavior remains nascent. To unravel the details of complex nervous system function, we must consider not only the morphological and physiological properties of individual neurons, but also the structure and function of connections formed between different cell types. In the last decade much effort has been focused on trying to fully characterize “the brain connectome” and to understand how patterns of synaptic connectivity between neurons might help to better inform the underlying defects associated with neurological and psychiatric disorders (Sporns et al., 2005; Lichtman and Sanes, 2008). More recently there is a growing interest in mapping, and eventually classifying, all synapses in the brain to construct a complete “synaptome” (DeFelipe, 2010; O'Rourke et al., 2012). The very nature of these studies, which rely on multidisciplinary research efforts, have thus catalyzed the development of new research tools and technologies. For instance, advances in molecular genetics, viral engineering, and imaging technologies now allow precise labeling, manipulation, and mapping of complex neural circuits, together revealing previously unattainable details about the cellular morphologies and subcellular structures that are unique to the different types of neurons that make up the brain. Such technological advances could not be possible without successful co-evolution of novel computational tools and analytical methods that allow acquisition, management, and interpretation of gigantic and complex datasets. In fact, this last point perhaps represents the main challenge for the future of “connectomics” (Lichtman et al., 2014).

This Research Topic comprises a wide variety of articles contributing to current views and understanding of different neural circuits, how they are organized in neural networks, and what are the functional outputs of this organization. Additionally, pioneering researchers in the field review novel high-throughput tools and analytical approaches, further describing how these methods have evolved to better explore neural circuits at different levels, covering a wide spectrum of analyses that range from the study of big volumes of brain tissue, to the functional properties of a given network.

The Research Topic eBook is organized into three chapters that cover different aspects of our current knowledge of neural circuits. In chapter 1 the reader will find articles related to the architecture and structural definition of neural circuits. In this chapter Li et al. (2014) use a combination of classical immunofluorescence techniques and immuno-Electron Microscopy (EM) to understand the neuronal connectivity of a pain-related circuitry. With a similar aim, Serrano-Velez et al. (2014) apply fluorescent retrograde labeling together with freeze-fracture replica immunogold labeling (FRIL) to understand the synaptic organization of the mosquitofish spinal cord. Marc et al. (2014) fully characterized the complete connectome of a retinal cell population using a combination of serial-EM and immunolabeling of small molecules. Soiza-Reilly and Commons (2014) discuss recent evidence about the complex architecture of the dorsal raphe's synaptic neuropil using the novel high-resolution imaging technique called array tomography. Nagayama et al. (2014) review the wiring diagram of the olfactory bulb integrating anatomical and functional aspects. In chapter 2, authors describe and discuss genetic and chemical manipulations of neuronal populations to understand their functionality and their role in neural networks. Thus, Harris et al. (2014) present a high-throughput characterization of numerous new mouse lines that express Cre recombinase selectively in different neuronal populations, allowing dissection and manipulation of neuronal population-specific circuits. Burgalossi et al. (2014) explore the complex neural coding of the medial entorhinal cortex under different environmental conditions. Rothermel and Wachowiak (2014) use novel genetically-encoded calcium sensors (GCaMPs) to explore the cortical feedback to the olfactory bulb. Kawashima et al. (2014) review recently characterized activity-dependent promoters and enhancer elements particularly useful for monitoring the activity of neural networks. Additionally, Murphey et al. (2014) discuss how genetically encoded fluorescent proteins, viral tracing methods, opto- and chemical genetics, and biosensors can be used in the study of inhibitory interneurons. Finally, in chapter 3, new methods and tools for data acquisition and analysis are introduced and discussed. Mazurek et al. (2014) describe new analytical tools to extract quantitative data from orientation and direction selectivity studies in the visual cortex. Hayworth et al. (2014) introduce a novel approach for mapping and imaging large libraries of ultrathin sections. In addition, Lütcke et al. (2013) present a novel method to assess the quality of spike dynamics and network topology inferred from calcium imaging data.

Together, this Research Topic brings to the readers not only new neurobiological data and novel analytical tools, but also offers new perspectives about the way we think about neural circuits and networks, giving rise to important insights to be considered when exploring structural and functional features of micro- and macrocircuits.

## Conflict of interest statement

The authors declare that the research was conducted in the absence of any commercial or financial relationships that could be construed as a potential conflict of interest.

## References

[B1] Arenkiel B. R. Tvrdik P. Gaufo G. O. Capecchi M. R. (2004). Hoxb1 functions in both motoneurons and in tissues of the periphery to establish and maintain the proper neuronal circuitry. Genes Dev. 18, 1539–1552. 10.1101/gad.1207204 15198977 PMC443517

[B2] Burgalossi A. von Heimendahl M. Brecht M. (2014). Deep layer neurons in the rat medial entorhinal cortex fire sparsely irrespective of spatial novelty. Front. Neural Circuits 8:74. 10.3389/fncir.2014.00074 25071455 PMC4092364

[B3] Dasen J. S. (2009). Transcriptional networks in the early development of sensory-motor circuits. Curr. Top. Dev. Biol. 87, 119–148. 10.1016/S0070-2153(09)01204-6 19427518

[B4] DeFelipe J. (2010). From the connectome to the synaptome: an epic love story. Science 330, 1198–1201. 10.1126/science.1193378 21109663

[B5] Harris J. A. Hirokawa K. E. Sorensen S. A. Gu H. Mills M. Ng L. L.. (2014). Anatomical characterization of Cre driver mice for neural circuit mapping and manipulation. Front. Neural Circuits 8:76. 10.3389/fncir.2014.00076 25071457 PMC4091307

[B6] Hayworth K. J. Morgan J. L. Schalek R. Berger D. R. Hildebrand D. G. C. Lichtman J. W. (2014). Imaging ATUM ultrathin section libraries with WaferMapper: a multi-scale approach to EM reconstruction of neural circuits. Front. Neural Circuits 8:68. 10.3389/fncir.2014.00068 25018701 PMC4073626

[B7] Inamata Y. Shirasaki R. (2014). Dbx1 triggers crucial molecular programs required for midline crossing by midbrain commissural axons. Development 141, 1260–1271. 10.1242/dev.102327 24553291

[B8] Kawashima T. Okuno H. Bito H. (2014). A new era for functional labeling of neurons: activity-dependent promoters have come of age. Front. Neural Circuits 8:37. 10.3389/fncir.2014.00037 24795570 PMC4005930

[B9] Lichtman J. W. Pfister H. Shavit N. (2014). The big data challenges of connectomics. Nat. Neurosci. 17, 1448–1454. 10.1038/nn.3837 25349911 PMC4412267

[B10] Lichtman J. W. Sanes J. R. (2008). Ome sweet ome: what can the genome tell us about the connectome? Curr. Opin. Neurobiol. 18, 346–353. 10.1016/j.conb.2008.08.010 18801435 PMC2735215

[B11] Li M.-Y. Wu Z.-Y. Lu Y.-C. Yin J.-B. Wang J. Zhang T.. (2014). Connections between EM2-containing terminals and GABA/μ-opioid receptor co-expressing neurons in the rat spinal trigeminal caudal nucleus. Front. Neural Circuits 8:125. 10.3389/fncir.2014.00125 25386121 PMC4208411

[B12] Lütcke H. Gerhard F. Zenke F. Gerstner W. Helmchen F. (2013). Inference of neuronal network spike dynamics and topology from calcium imaging data. Front. Neural Circuits 7:201. 10.3389/fncir.2013.00201 24399936 PMC3871709

[B13] Marc R. E. Anderson J. R. Jones B. W. Sigulinsky C. L. Lauritzen J. S. (2014). The AII amacrine cell connectome: a dense network hub. Front. Neural Circuits 8:104. 10.3389/fncir.2014.00104 25237297 PMC4154443

[B14] Mazurek M. Kager M. Van Hooser S. D. (2014). Robust quantification of orientation selectivity and direction selectivity. Front. Neural Circuits 8:92. 10.3389/fncir.2014.00092 25147504 PMC4123790

[B15] Murphey D. K. Herman A. M. Arenkiel B. R. (2014). Dissecting inhibitory brain circuits with genetically-targeted technologies. Front. Neural Circuits 8:124. 10.3389/fncir.2014.00124 25368555 PMC4201106

[B16] Nagayama S. Homma R. Imamura F. (2014). Neuronal organization of olfactory bulb circuits. Front. Neural Circuits 8:98. 10.3389/fncir.2014.00098 25232305 PMC4153298

[B17] O'Rourke N. A. Weiler N. C. Micheva K. D. Smith S. J. (2012). Deep molecular diversity of mammalian synapses: why it matters and how to measure it. Nat. Rev. Neurosci. 13, 365–379. 10.1038/nrn3170 22573027 PMC3670986

[B18] Pecho-Vrieseling E. Sigrist M. Yoshida Y. Jessell T. M. Arber S. (2009). Specificity of sensory-motor connections encoded by Sema3e-Plxnd1 recognition. Nature 459, 842–846. 10.1038/nature08000 19421194 PMC2847258

[B19] Rothermel M. Wachowiak M. (2014). Functional imaging of cortical feedback projections to the olfactory bulb. Front. Neural Circuits 8:73. 10.3389/fncir.2014.00073 25071454 PMC4080262

[B20] Serrano-Velez J. L. Rodriguez-Alvarado M. Torres-Vazquez I. I. Fraser S. E. Yasumura T. Vanderpool K. G.. (2014). Abundance of gap junctions at glutamatergic mixed synapses in adult Mosquitofish spinal cord neurons. Front. Neural Circuits 8:66. 10.3389/fncir.2014.00066 25018700 PMC4072101

[B21] Soiza-Reilly M. Commons K. G. (2014). Unraveling the architecture of the dorsal raphe synaptic neuropil using high-resolution neuroanatomy. Front. Neural Circuits 8:105. 10.3389/fncir.2014.00105 25206323 PMC4143723

[B22] Sporns O. Tononi G. Kötter R. (2005). The human connectome: a structural description of the human brain. PLoS Comput. Biol. 1:e42. 10.1371/journal.pcbi.0010042 16201007 PMC1239902

[B23] Sürmeli G. Akay T. Ippolito G. C. Tucker P. W. Jessell T. M. (2011). Patterns of spinal sensory-motor connectivity prescribed by a dorsoventral positional template. Cell 147, 653–665. 10.1016/j.cell.2011.10.012 22036571 PMC3238499

[B24] White J. J. Sillitoe R. V. (2013). Development of the cerebellum: from gene expression patterns to circuit maps. Wiley Interdiscip. Rev. Dev. Biol. 2, 149–164. 10.1002/wdev.65 23799634

